# Supplementary material for: Emergency department care experiences among youth with mental health concerns
Source: PLOS Ment Health. 2024 Dec 31;1(7):e0000200. doi: 10.1371/journal.pmen.0000200 (PMC12798253; doi:10.1371/journal.pmen.0000200)
Supplement: S1 Table — Full list of self-interpretation dyad and triad questions with possible responses, all of which were optional to respond to. (PDF) [file pmen.0000200.s001.pdf]

**Question**	**Possible Responses**
Dyads	*Response plotted between 2 options*
S1. During the emergency room visit, the patient's personal situation, identity, or culture received…	1) Far too little attention; 2) Far too much attention
S2. The events in the story were mostly about…	1) How the hospital works; 2) Emergency room staff
S3. Based on the story shared, the patient's ability to pay for care or other costs (medicines, travel for care, etc.) received…	1) Far too much attention; 2) Far too little attention
S4. Based on the story shared, the patient was given…	1) Too little attention to their needs; 2) Too much attention to their needs
S5. During the visit, how much control/say did the patient have in making decisions about their care	1) Too much control; 2) Too little control
S6. In the experience shared, it was more important for the patient to…	1) Receive the best possible medical care; 2) Be treated with kindness and respect
Triads	*Response plotted between 3 options*
T1. During the events in the shared story, the patient was…	1) Judged; 2) Powerless/not in control; 3) Ignored
T2. During this story, the patient was…	1) Empowered/in control; 2) Informed; 3) Accepted/valued
T3. In the shared experience, the doctors, nurses and other emergency room staff…	1) Understood the situation; 2) Showed they cared; 3) Shared important information
T4. The patients shared experience was affected most by:	1) How emergency room staff behaved towards the patient; 2) Medical care/testing provided; 3) Wait times
T5. After the patient left the emergency room, he/she was:	1) Not sure of what to do next; 2) Unclear about their health condition; 3) Unsupported in coping with their concern

T6. Based on the shared story, the following would improve future emergency room care:	1) Better understanding of personal situation, identity and culture; 2) Easier access to medical care; 3) Better communication between health care workers
