# Supplementary material for: Emergency department care experiences among youth with mental health concerns
Source: PLOS Ment Health. 2024 Dec 31;1(7):e0000200. doi: 10.1371/journal.pmen.0000200 (PMC12798253; doi:10.1371/journal.pmen.0000200)
Supplement: S2 Table — Full list of survey multiple-choice questions with possible responses. (PDF) [file pmen.0000200.s002.pdf]

Question	Possible Responses
Questions about the story shared	
Who was the patient the story (choose only 1)?	• It was me<br>• It was my child<br>• It was my parent/guardian<br>• It was my spouse/partner<br>• It was someone else in my family<br>• It was a friend<br>• It was a person for whom I am a caregiver (paid or volunteer)<br>• Other<br>• Not sure/prefer not to say
Was the visit in your shared story for concerns related to COVID (choose only 1)?	• Yes<br>• No<br>• Not sure/prefer not to say
In what way did COVID change how easy it was for the patient to get care in the shared story (choose only 1)?	• It was easier to get the care needed<br>• It was harder to get the care needed<br>• COVID did not impact the patient's access to care<br>• Not sure/prefer not to say
How did COVID impact the patient's care experience in the story told (choose only 1)?	• The patient's experience was better than normal<br>• The patient's care experience was worse than normal<br>• COVID did not impact the patient's care experience<br>• Not sure/prefer not to say
How did you feel about the patient's visit overall (choose only 1)?	• Very bad<br>• Bad<br>• Not good or bad<br>• Good<br>• Very good<br>• Not sure/prefer not to say
Some groups can face barriers to accessing health care. Which of the following, if any, most relates to the patient in the story (choose up to 3)?:	• Ethnic minority/person of colour<br>• Indigenous<br>• Person with a disability<br>• Mental health concern or illness<br>• 2SLGBTQ+<br>• Alcohol/drugs or substance use<br>• Homelessness/without stable housing<br>• Trading sex for food, money or goods<br>• Member of a gang<br>• Incarceration (in jail/prison)<br>• Sexual assault or experiencing violence<br>• None of the above<br>• Other<br>• Not sure/prefer not to say
How did the patient's personal situation, identity, and/or culture affect the experience (choose only 1)?	• In a very bad way<br>• In a bad way<br>• It did not affect the treatment received<br>• In a good way

	• In a very good way<br>• Not sure/prefer not to say
If the patient identifies as Indigenous, which Indigenous group do they belong (choose only 1)?:	• Patient is not Indigenous<br>• First Nations<br>• Metis<br>• Inuit<br>• Other
If the patient is a person with a disability, which relates most to the story shared (choose only 1)?	• Patient is not a person with a disability<br>• Physical disability<br>• Mental health disability<br>• Low vision/blindness<br>• Hearing loss/deafness<br>• Intellectual disability<br>• Other<br>• Not sure/prefer not to say
What is the patient's ethnicity (choose only 1)?	• Indigenous<br>• White/European<br>• South Asian<br>• Chinees<br>• Black<br>• Filipino<br>• Latin American<br>• Arab<br>• Southeast Asian<br>• West Asian<br>• Korean<br>• Japanese<br>• One or more ethnicity<br>• Other<br>• Not sure/prefer not to say
How does the patient identify (choose only 1)?	• Man<br>• Woman<br>• Non-binary<br>• Not sure/prefer not to say
What is the patient's sexual orientation (choose only 1)?	• Straight<br>• Gay/Lesbian<br>• Bisexual<br>• Pansexual<br>• Asexual<br>• Questioning/unsure<br>• Not on this list<br>• Not sure/prefer not to say
Does the patient identify as gender diverse (transgender, two-spirt, gender-fluid, non-binary, or agender) (choose only 1)?	• Yes<br>• No<br>• Not sure/prefer not to say
Was the patient in your story (choose only 1)?	• <18 years of age<br>• 18-25 years of age<br>• 26-45 years of age<br>• 46 – 65 years of age<br>• >65 years of age<br>• Not sure/prefer not to say

How often does the patient in your story struggle to make ends meet (not enough money for food, bills, housing, clothes, etc.) (choose only 1)?	• Never<br>• Rarely<br>• Sometimes<br>• Often<br>• All the time<br>• Not sure/prefer not to say
How long ago did the shared emergency room visit occur? (choose only 1)?	• 0-6 months<br>• 7-12 months<br>• 13-18 months<br>• 19-24 months<br>• More than 24 months<br>• Not sure/prefer not to say
How often did the patient in the story go to the emergency room in Kingston in the 24 months prior to the visit shared (choose only 1)?	• Did not access care in the emergency room before the experience described<br>• 1-3 times<br>• 4-6 times<br>• 7-9 times<br>• 10 or more times<br>• Not sure/prefer not to say
The events in the story were mostly focused on which part of the emergency room (choose only 1)?	• Triage (where nurse took your blood pressure)<br>• Registration (where a clerk registered your health care)<br>• Waiting room<br>• Nursing staff<br>• Doctors<br>• Social workers<br>• Porters, x-ray and/or CAT scan technicians<br>• Security officers<br>• Discharge<br>• Other<br>• Not sure/prefer not to say
How often do you think the situation in your story occurs (choose only 1)?	• It is very rare<br>• It happens from time to time<br>• It is somewhat typical<br>• It happens all the time<br>• Not sure/prefer not to say
How did your story make you feel at the time it occurred (chose up to 3)?	• Accepted<br>• Afraid<br>• Disappointed<br>• Embarrassed<br>• Frustrated<br>• Happy<br>• Hopeful<br>• Relieved<br>• Satisfied<br>• Thankful<br>• Worried<br>• Not sure/prefer not to say
Is this story about the patient being treated without respect or the patient being judged (choose only 1)?	• Yes<br>• No<br>• Not sure/prefer not to say
Questions about person completing survey	

What is your age (choose only 1)?	• 16-24<br>• 25-34<br>• 35-44<br>• 45-54<br>• 55-64<br>• 64 or older<br>• Not sure/prefer not to say
What is your ethnicity (choose only 1)?	• Indigenous<br>• White/European<br>• South Asian<br>• Chinese<br>• Black<br>• Filipino<br>• Latin American<br>• Arab<br>• Southeast Asian<br>• West Asian<br>• Korean<br>• Japanese<br>• One or more ethnicity<br>• Other<br>• Not sure/prefer not to say
How do you identify (choose only 1)?	• Man<br>• Woman<br>• Non-binary<br>• Prefer not to say
Where did you learn about this study (choose only 1)?	• Kingston General Hospital Emergency Department<br>• Hotel Dieu Hospital Urgent Care Centre<br>• Word of mouth<br>• Shared online through social media<br>• Other organization

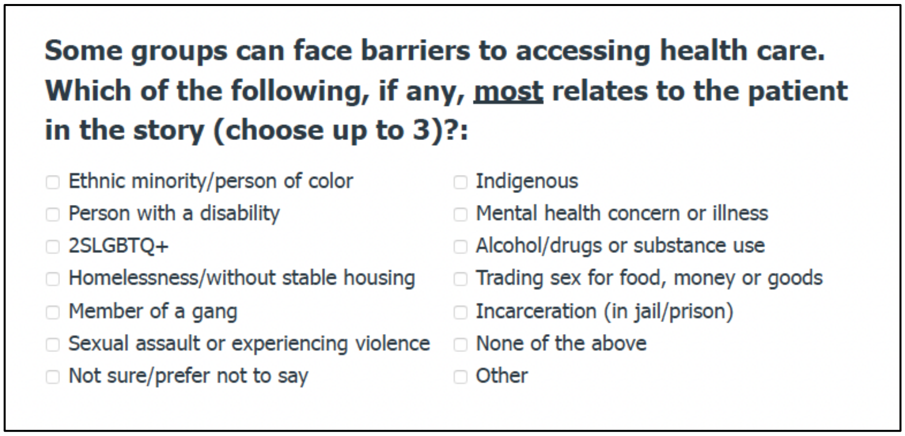

Some groups can face barriers to accessing health care. Which of the following, if any, most relates to the patient in the story (choose up to 3)?:

- ☐ Ethnic minority/person of color
- ☐ Person with a disability
- ☐ 2SLGBTQ+
- ☐ Homelessness/without stable housing
- ☐ Member of a gang
- ☐ Sexual assault or experiencing violence
- ☐ Not sure/prefer not to say
- ☐ Indigenous
- ☐ Mental health concern or illness
- ☐ Alcohol/drugs or substance use
- ☐ Trading sex for food, money or goods
- ☐ Incarceration (in jail/prison)
- ☐ None of the above
- ☐ Other

S2 Figure. Equity deserving identity survey question. Excerpt of survey question used to identify study comparison groups.
