# Supplementary material for: Emergency department care experiences among youth with mental health concerns
Source: PLOS Ment Health. 2024 Dec 31;1(7):e0000200. doi: 10.1371/journal.pmen.0000200 (PMC12798253; doi:10.1371/journal.pmen.0000200)
Supplement: S1 Fig — Example of triad (left) and dyad (right) self-interpretation questions from survey through Spryng.io (23). (PDF) [file pmen.0000200.s005.pdf]

Example of a Triad Question:

During this story, the patient was....

Empowered/in control

Informed

Accepted/valued

☐ This question does not relate to the story I shared/I do not want to answer

Example of a Dyad Question:

The events in the story were mostly about...

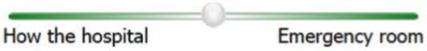

How the hospital works

Emergency room staff

☐ This question does not relate to the story I shared/I do not want to answer
