# Supplementary material for: Emergency department care experiences among youth with mental health concerns
Source: PLOS Ment Health. 2024 Dec 31;1(7):e0000200. doi: 10.1371/journal.pmen.0000200 (PMC12798253; doi:10.1371/journal.pmen.0000200)
Supplement: S2 Fig — Excerpt of survey question used to identify study comparison groups. (PDF) [file pmen.0000200.s006.pdf]

Some groups can face barriers to accessing health care. Which of the following, if any, most relates to the patient in the story (choose up to 3)?:

- ▢ Ethnic minority/person of color
- ▢ Indigenous
- ▢ Person with a disability
- ▢ Mental health concern or illness
- ▢ 2SLGBTQ+
- ▢ Alcohol/drugs or substance use
- ▢ Homelessness/without stable housing
- ▢ Trading sex for food, money or goods
- ▢ Member of a gang
- ▢ Incarceration (in jail/prison)
- ▢ Sexual assault or experiencing violence
- ▢ None of the above
- ▢ Not sure/prefer not to say
- ▢ Other
