# Supplementary material for: Emergency department care experiences among youth with mental health concerns
Source: PLOS Ment Health. 2024 Dec 31;1(7):e0000200. doi: 10.1371/journal.pmen.0000200 (PMC12798253; doi:10.1371/journal.pmen.0000200)
Supplement: Attachment — Submitted filename: Response to Reviewers.docx [file pmen.0000200.s007.docx]

September 18, 2024

Dr. Rachel Dale, PhD
Academic Editor
PLOS Mental Health

Dear Dr. Rachel Dale, Dr. Rebecca Appleton, and Dr. Vania Martinez,

Thank you for taking the time to provide thoughtful feedback on our manuscript. We have reviewed your comments and suggestions and incorporated as they are described below in the document following this letter. Our responses to your comments and suggestions are indicated in blue for ease of review.

Thank you again for your time and efforts. We look forward to hearing back after you review our revised manuscript.

Yours sincerely,

Laura Wells, MD

Primary Author

Resident Physician

Department of Family Medicine

Queen’s University, Kingston, ON, Canada

Melanie Walker, PhD

Corresponding Author

Research Scientist/Assistant Professor

Depts. Emergency Medicine/Public Health Sciences

Queen’s University, Kingston, ON, Canada

**Journal Requirements**

1. Please review your reference list to ensure that it is complete and correct. If you have cited papers that have been retracted, please include the rationale for doing so in the manuscript text, or remove these references and replace them with relevant current references. Any changes to the reference list should be mentioned in the rebuttal letter that accompanies your revised manuscript. If you need to cite a retracted article, indicate the article’s retracted status in the References list and also include a citation and full reference for the retraction notice.

The authors have reviewed the reference list and all references are complete and correct. There are no papers cited that have been retracted. There were three new references added as suggested in the comments below, and the reference list numbering in-text and in the reference list have been updated to reflect these additionally cited papers. The final reference list was reviewed and edited to ensure proper formatting for the revised manuscript. Page numbers were also added to the manuscript.

**Editor**

Comments to the Author

Thanks to the authors for this paper. The reviewers and I suggest some minor revisions prior to publication. Please pay particular attention to the reviewer’s comments regarding the use if abbreviations and the term ‘equity-deserving’.

Thank you for taking the time to review this manuscript and to provide comments to improve the work.

General

1. In general, there is an extensive use of abbreviations that can make the manuscript a bit difficult to follow. For example on line 76, to what does EM refer? Additionally line 102, KHSC ED/UCC is a new abbreviation and since it is likely only used rarely, can perhaps be written out in full. Line 219: HCP?

Thank you for this suggestion. We have removed the abbreviations you highlighted that are used infrequently and instead have written out in full. Further, we have ensured all new abbreviations have first been introduced within the text before use.

2. Lines 200-201: here both sex and gender are referred to. Therefore I just want to check that in the focus groups participants were categorised according to sex and in the survey they were categorised according to gender? If not then this should be corrected such that female/male/intersex refer to sex and man/woman/gender-diverse refer to gender.

We appreciate you pointing this out. We collected gender (not sex) in the study survey; We did not formally collect gender nor sex in the focus group discussions but gender was collected informally through self-identification. We have corrected the language to properly reflect that focus group participants were also categorized according to gender based on self-identification in the revised manuscript on what is now page 10, line 220.

Results

3. Please report full statistics rather than just the p-values.

Thank you for this suggestion. The inferential statistics have been added to table 1 and table 2 on pages 10-13 to reflect this.

4. Table 2: how is it possible that 29 participants had no ED visit in the prior 2 years when on line 106 it states that this is part of the inclusion criteria? As I understand it the purpose of this study is to assess people with direct ED visit experience (lines 124-125) so these 29 participants should have been excluded.

Thank you for the astute observation. The language for this survey question was clarified in Table 2 on page 12, line 242 of the revised manuscript. Participants were asked to identify how many times they visited a Kingston ED in the 24 months prior to the visit shared, which is separate from the study inclusion criteria of sharing a recent (within the last 24 months) ED visit experience.

Minor Points

5. Line 69-70: some commas are missing - and improve YMHC experiences with, and interaction with, the health system,…

Thank you for identifying this – the grammar has been corrected in this sentence on what is now page 4, line 71.

6. Line 140: The Chi-squared test is an inferential statistic.

Thank you for identifying this, we have changed the language on page 7, lines 153, 156, and 158 in the revised manuscript to include this.

7. Line 365: for suicidal behaviour? suicidal ideation?

Thank you for this note. Participants did not use the term suicidal behaviour or suicidal ideation in this context, and thus this was not reported as such. However, we have added to the text on page 20, lines 392-393 to identify that participants noted mental health, trauma, and being unhoused as exacerbating these stresses to make this clearer.

8. Line 375: perhaps trauma-informed care could be very briefly explained.

Thank you for this suggestion. We have incorporated a brief definition of trauma-informed care where it is mentioned in the discussion on page 21, lines 416-422:

*Trauma-informed care involves acknowledging the impact and signs of trauma in an individual, and integrating this knowledge to provide appropriate care for an individual that avoids re-traumatization. There are a set of core principles that are integral to providing both individual and organizational trauma-informed care, which include safety, trustworthiness and transparency, peer support, collaboration, empowerment, and humility and responsiveness (35).*

References added to address this suggestion include:

35. Centre for Health Care Strategies. What is trauma-informed care? [Internet] Centre for Health Care Strategies; 2024 [cited 2024 Aug 29]. Available from: <https://www.traumainformedcare.chcs.org/what-is-trauma-informed-care/>.

**Reviewer 1**

Comments to the Author

Thanks for the opportunity to review this paper. It adds to existing evidence regarding the need for improving ED support for young people with mental health problems.
There are some points which require amendment or clarification before publication.

Thank you for your review and suggestions, which improve upon this work.

Abstract

1. What do you mean by 'equity deserving'? - suggest leaving this terminology out if there isn't space to define it.

Thanks for this suggestion. We have added in a brief definition in the abstract and methods to make this clearer in the abstract on page 2, lines 31-33: “*Equity deserving groups are defined as individuals who face shared barriers to participating in society and care as a result of identifying with a certain community.”* This is based on a common accepted definition across various Health Equity Offices, such as the Queen’s University Human Rights and Equity Office.

2. Can you add something to the methods section about how you collected qualitative data? It's not clear if this was interviews, a qual survey etc

Thank you for seeking clarification. In the Methods section on page 6, lines 118-134, we have described the sensemaking data collection survey tool used called Spryng.io that allowed us to capture both qualitative and quantitative ED care experiences from study participants. We have added some further text to the section on qualitative analysis on page 8, lines 168-169 to make this clearer where the qualitative data was derived from.

Introduction

3. Line 83 - The last sentence of your introduction doesn't make sense

Thank you for pointing this out. We have removed this sentence from the text and subsequently elaborated on rationale for study as outlined in the response to point 4 below on page 4, lines 84-88.

4. In general I think the rationale for why the present research is needed could be made stronger. You have presented lots of previous research in this area already - why exactly is your study needed and how does it build on what has gone before?

Thank you for your comment. Mainly, it acts to expand on existing qualitative literature with added quantitative findings to increase generalizability. Additionally, comparing the experiences of youth with a MH concern with an age-matched control allows the identification of experiences attributable to identifying as having a MH concern. We have added supporting text at the end of the introduction one page 4, lines 85-89 to better explain and justify the rationale of our study, and the gap it fills.

Methods

5. Is it correct that young people didn't need to have visited the ED with a concern relating to their mental health to be in the EDG group? please make this explicit in the text if so.

Thank you for suggesting this, you are correct in your statement. The reason for this is to identify if youth with a MH concern experienced differences in care in the ED as it pertains to their MH identity, regardless of if the visit was for a MH concern. We have added a few sentences in the Study Design and Data Source section of the methods on page 6, lines 123-125 to make this clearer. There is also a sentence on this the section on Selection of Participants on page 7, lines 140-141 to ensure this is also explicit as it relates to the sub-analysis among youth.

6. How are you defining 'micronarratives'?

Thank you for identifying this. The term has been used to express that the qualitative shared experiences are briefer than traditional qualitative transcripts and thus is used as a better descriptor. We have added an explanation and definition to this effect on page 8, lines 168-172.

7. Your primary outcome of 'ED care experiences' isn't really an outcome - can you make this more specific?

Thank you for this suggestion. We have re-phrased this in the methods section on page 7, line 148-150 to: *“The primary outcome of this study was to identify how YMHC experienced ED care, and if those experiences differed compared to youth without MH concerns*.”

8. You should add references for the steps in your qualitative analysis.

Thank you for suggesting this. We have identified relevant references to support our approach to qualitative analysis and included these in the manuscript on page 8, lines 172-182.

References added to address this comment include:

29. Thomas J, Harden A. Methods for the thematic synthesis of qualitative research in systematic reviews. BMC Medical Research Methodology. 2008;8(1):45.

30. Collins SE, Clifasefi SA-O, Stanton J, The Leap Advisory B, Straits KA-O, Gil-Kashiwabara EA-O, et al. Community-based participatory research (CBPR): Towards equitable involvement of community in psychology research. (1935-990X (Electronic)).

Results

9. Can you add some information as participant identifiers after the quotes? This might read better than introducing each quote by participant characteristics.

Thank you for suggesting this idea. Where appropriate we have changed select quotes to add patient identifiers after the quotes to improve readability on page 14, lines 268-275; page 16, lines 303-308; pages 17-18, lines 341-345 and 349-354; and pages 19-20, lines 375-380 and 385-390.

**Reviewer 2**

Comments to the Author

The study is well presented and is very interesting for the mixed methodology used and its most relevant findings as well as for the proposals for improvement.

Thank you for your comments and reviewing this work.

1. Please review the use of the abbreviation MH, so that it is explained the first time it is used.

Thank you for identifying and suggesting this. We have edited the text to ensure this abbreviation, as well as all other abbreviations in the text, are defined when first used (page 3, lines 50-51).

2. Please explain why you included in the sample people who had no ED visits in the last 2 years (table2).

Thank you for the important observation. The language for this survey question was clarified in Table 2 on page 12, line 242 of the revised manuscript. Participants were asked to identify how many times they visited a Kingston ED in the 24 months prior to the visit shared, which is separate from the study inclusion criteria of sharing a recent (within the last 24 months) ED visit experience.
