# Supplementary material for: Emergency department care experiences among youth with mental health concerns
Source: PLOS Ment Health. 2024 Dec 31;1(7):e0000200. doi: 10.1371/journal.pmen.0000200 (PMC12798253; doi:10.1371/journal.pmen.0000200)
Supplement: Attachment — Submitted filename: Response to Reviewers_Nov 12 2024.docx [file pmen.0000200.s008.docx]

November 11^th^, 2024

Dr. Rachel Dale, PhD
Academic Editor
PLOS Mental Health

Dear Dr. Rachel Dale, Dr. Rebecca Appleton, and Dr. Vania Martinez,

Thank you for taking the time to review our responses to your comments. We have reviewed your most recent suggestions and incorporated them as they are described below in the document following this letter. Our responses to your comments and suggestions are indicated in blue for ease of review.

Thank you again for your time. We look forward to hearing back after you review our most recently revised manuscript.

Yours sincerely,

Laura Wells, MD

Primary Author

Resident Physician

Department of Family Medicine

Queen’s University, Kingston, ON, Canada

Melanie Walker, PhD

Corresponding Author

Research Scientist/Assistant Professor

Depts. Emergency Medicine/Public Health Sciences

Queen’s University, Kingston, ON, Canada

**Editor**

Comments to the Author

The reviewers and I agree the revision has addressed all comments. Thanks to the authors for their responses!

As a final minor edit, it has come to my attention that PLOS Mental Health has specific guidelines for reporting qualitative research:

PLOS Mental Health considers qualitative and mixed-methods studies for publication. We recommend that authors use the Consolidated criteria for reporting qualitative research (COREQ) checklist or Standards for reporting qualitative research (SRQR) checklist. ([http://journals.plos.org/plosmentalhealth/s/submission-guidelines#loc-qualitative-research](https://can01.safelinks.protection.outlook.com/?url=https%3A%2F%2Furldefense.com%2Fv3%2F__http%3A%2F%2Fjournals.plos.org%2Fplosmentalhealth%2Fs%2Fsubmission-guidelines*loc-qualitative-research__%3BIw!!OXY76JYTP54u!F5Hg_UEUggRPeFPDp2jjyANxfF0OvPg4LfNTwkfL48kIL6FN7RY7dPsGvk4q8umndvyG41kOzvp1z2yI1NNuQKLsmw%24&data=05%7C02%7Claura.kwells%40queensu.ca%7C00a597df40104183689408dcfdb7c8ef%7Cd61ecb3b38b142d582c4efb2838b925c%7C1%7C0%7C638664213015718452%7CUnknown%7CTWFpbGZsb3d8eyJFbXB0eU1hcGkiOnRydWUsIlYiOiIwLjAuMDAwMCIsIlAiOiJXaW4zMiIsIkFOIjoiTWFpbCIsIldUIjoyfQ%3D%3D%7C0%7C%7C%7C&sdata=M0vJuIcmvfRHuSmeNM5TUaPgD6zg5r2awVCkvJXr95c%3D&reserved=0)).

Please check that your reporting follows the items of one of the above checklists and state in the methods section which checklist was adhered to. Apologies for not including this in the original review. However, I believe the manuscript mostly follows the checklist criteria so I hope this will not be too much work.

We appreciate you pointing this out and directing us to the appropriate reporting checklists. We have completed the COREQ checklist and included this as a supplementary document (see S3 Table). We have also added a sentence in the text to reference that this checklist was adhered to and completed on page 8, lines 167-168. Additionally, we have also made minor edits to ensure it is clear how we have adhered to the checklist as follows:

Page 5, line 112: Clarified that a trained research assistant administered the survey.

Page 6, line 119 – 120: clarified that the survey was completed via electronic tablet.

Page 8, lines 172 – 173: clarified that Microsoft Excel was used for coding.

Page 10, lines 214 – 217: included the number of people who declined to participate and reasons for not participating from the parent study.

Page 30, line 624-625: Added the COREQ checklist to the list of supplementary documents.
